# Effects of Ion-Releasing Materials on Dentine: Analysis of Microhardness, Appearance, and Chemical Composition

**DOI:** 10.3390/ma16237310

**Published:** 2023-11-24

**Authors:** Ivan Šalinović, Falk Schwendicke, Haitham Askar, Jamila Yassine, Ivana Miletić

**Affiliations:** 1 Department for Endodontics and Restorative Dentistry, School of Dental Medicine, University of Zagreb, Gundulićeva 5, 10000 Zagreb, Croatia; miletic@sfzg.hr; 2 Department of Oral Diagnostics, Digital Health and Health Services Research, Charité—Universitätsmedizin Berlin, Aßmannshauser Straße 4-6, 14197 Berlin, Germany; falk.schwendicke@charite.de; 3 Department of Operative, Preventive and Pediatric Dentistry, Charité—Universitätsmedizin Berlin, Aßmannshauser Straße 4-6, 14197 Berlin, Germany; haitham.askar@charite.de; 4 Department for Dental Prosthetics, Geriatric Dentistry and Functional Theory, Charité—Universitätsmedizin Berlin, Aßmannshauser Straße 4-6, 14197 Berlin, Germany; jamila.yassine@charite.de

**Keywords:** dentine, ion-releasing materials, microhardness, glass-ionomer cements, glass hybrids, alkasite

## Abstract

The aim of this study was to compare the potential of standard ion-releasing materials to repair demineralized lesions with recently introduced alkasite and glass hybrid materials. Glass ionomer (GC Fuji TRIAGE), two glass hybrids (EQUIA Forte HT, Riva SC), calcium silicate cement (Biodentine) and an alkasite (Cention Forte) were tested. A total of 72 human third molars were used for sample preparation; on the dentine surface, a class-I cavity was prepared, and one half was covered with nail varnish. The teeth were subjected to a demineralization protocol, filled with the examined materials, and cut in half. The evaluation included a dentine microhardness assessment (*n* = 10) and SEM/EDS analysis (*n* = 2). The results were analyzed using SPSS 22.0 statistical software and compared using an analysis of variance and Scheffe post-hoc test. The statistical significance level was set to 0.05. Mean microhardness values (HV0.1) after 14 and 28 days were, respectively: EQUIA Forte HT (26.7 ± 1.45 and 37.74 ± 1.56), Riva Self Cure (19.66 ± 1.02 and 29.58 ± 1.18), Cention Forte (19.01 ± 1.24 and 27.93 ± 1.33), Biodentine (23.35 ± 1.23 and 29.92 ± 1.02), GC Fuji TRIAGE (25.94 ± 1.35 and 33.87 ± 5.57) and control group (15.57 ± 0.68 and 15.64 ± 0.82). The results were significantly different between most groups (*p* < 0.001). SEM/EDS revealed varying patterns, material deposits and distinct elemental variations. To conclude, all materials increased microhardness and affected the dentine surface appearance and chemical composition; EQUIA Forte HT demonstrated the most pronounced effects.

## 1. Introduction

Dentine formation is a complex process. It results in different types of dentine, each with its own characteristics [1]. Unlike enamel, dentine has a higher proportion of organic content, around 20%, most of which is collagen [2]. This complex organic matrix is what makes dentine remineralization a challenging process [3]. Demineralization of dentine, usually associated with the progression of a carious lesion, is initiated by a drop in pH. On the other hand, dentine remineralization requires the harmonious reparation of collagen and inorganic apatite, resulting in the intrafibrillar mineralization of collagen [4]. Furthermore, to restore the mechanical features of dentine, the processes of demineralization and remineralization need to be in synergistic connection to enable a precise mineral precipitation, both within the collagen intrafibrillar and interfibrillar spaces [5,6].

The contemporary approach to caries removal favors the usage of methods and materials that preserve hard dental tissue and promote its reparation, minimizing the risk of unintended pulp chamber exposure [7,8]. Additionally, previous research has shown that tissue preservation significantly improves the longevity of restorations [9]. Therefore, a growing emphasis has been placed on ion-releasing materials that elicit a specific biological response and bond to hard dental tissue, thus leading to tissue replacement that can reduce the susceptibility of tooth minerals to dissolution and/or is capable of restoring its attributes [6]. That group includes many different materials with various mechanisms of action. However, all of them are based on delivering ions into mineral-deprived lesions, aiming to repair them. Standard materials in this category are glass ionomer (GIC) and calcium silicate cements. Initially introduced in the 1970s [10], GICs have since been used in different areas of dental medicine due to their properties, which include biocompatibility, bioactivity, and fluoride release, making them efficient in promoting tissue repair and caries prevention [11]. However, their poor mechanical features and low resistance to wear and erosion have prevented them from becoming a long-term restorative material in permanent dentition [12,13,14,15]. To overcome these issues, glass hybrids have been introduced; they are reinforced by adding more reactive, smaller silica particle, and a higher molecular-weight acrylic and acid molecule, all of which increases the matrix cross-linking and improves their mechanical properties [16]. In addition, such materials also come with a resin-based coat, further improving their durability.

Before quick-setting modifications were developed, calcium silicate-based cements have mostly been used in endodontics, such as for the treatment of perforated roots, due to their high biocompatibility and ability to promote the formation of minerals [17]. Nowadays, some tri-calcium silicate-based cements are used as restorative materials and dentine replacements, as they induce mineral precipitation dentine formation [6]. While most of the previously listed materials are self-cured, alkasites, a group of recently introduced materials, are dual-cured. They contain an alkaline filler, thus releasing acid-neutralizing ions, as well as fluorides, hydroxyl ions and calcium in an acidic oral cavity environment [18].

Previous research has shown that the mechanical properties of dentin are best repaired when minerals are incorporated into the collagen fibrils, as the mere deposition of minerals into the demineralized lesion does not guarantee functional remineralization [19,20]. The present study assesses the biomechanical properties of dentine by testing microhardness and using scanning electron microscopy in conjunction with energy-dispersive X-ray spectroscopy (SEM/EDS).

We aimed to compare the potential of standard ion-releasing materials (glass ionomer and calcium silicate cements) to repair demineralized lesions with recently introduced alkasite and glass hybrids.

The null hypotheses were:

1. There is no difference in dentine microhardness values among the tested materials.
2. There are no differences in the mineral composition of the specimens treated with the tested materials.
3. There are no differences in the micro-surface appearance between groups.

## 2. Materials and Methods

The Ethics Committee of the School of Dental Medicine, University of Zagreb approved the protocol for the current study (05-PA-30-III-12/2021). The research was conducted at the University of Zagreb, School of Dental Medicine, the Ruđer Bošković Institute in Zagreb, the Faculty of Mechanical Engineering and Naval Architecture, University of Zagreb, and the Charite—Universitätsmedizin in Berlin. A total of 72 human third molars that were used in this study were collected at the Department of Oral Surgery, Clinical Hospital Centre Zagreb. Extracted teeth were thoroughly examined to ensure the absence of any carious lesions. Before use, the samples were placed in 0.5% chloramine solution at room temperature for up to three months.

### 2.1. Sample Preparation

Before the sample preparation, the teeth were meticulously cleaned using brushes, a scaler, and discs. The tested materials are listed in Table 1 (the type of material is provided by the manufacturer).

A non-bioactive composite material, 3M™ Filtek™ Universal Composite (3M ESPE, St. Paul, MN, USA), was used in the control group. The collected teeth were randomly divided into two groups for each of the five materials tested and a control group, as each of the two tests (after 14 and 28 days) used different specimens. Ten samples were obtained for each group in a single testing period for microhardness testing (*n* = 10). Two additional samples were prepared for SEM/EDS analysis in each group (*n* = 2). The occlusal third of the crown was removed using IsoMet 1000 Precision Cutter (Buehler, Lake Bluff, IL, USA) and IsoMet Diamond Wafering Blade (Buehler, Lake Bluff, IL, USA) at a speed of 200 rounds per minute, exposing the dentine surface. A class-I cavity with the floor ending at mid-coronal dentin (3 mm × 1.5 mm wide, 0.5 mm deep) was prepared in each tooth using a medium-grit (107 m) diamond bur (Komet, Lemgo, Germany) fixed in a water-cooled high-speed turbine. Half of the cavity was covered with acid-resistant nail polish (Markwins Beauty Brands, Inc., Walnut, CA, USA) to enable a direct comparison of surfaces. The samples were then demineralized by individually immersing them in a solution containing 0.0476 mM sodium fluoride (NaF), 2.2 mM calcium chloride dihydrate (CaCl_2_·2H_2_O), 2.2 mM potassium dihydrogen phosphate (KH_2_PO_4_), 50 mM acetic acid (CH_3_COOH), and 10 mM potassium hydroxide (KOH) at pH 5.0 (37 °C) for two weeks in an incubator ES 120 (NÜVE, Ankara, Turkey), as suggested by previous studies [22,23]. The cavities were then rinsed and air-dried, after which they were filled with one of the materials studied. All the materials were in encapsulated form and were mixed according to the manufacturer’s instructions in the Silver Mix capsule mixer (GC Corporation Tokyo, Japan); those treated with Cention Forte were further cured for 40 s with the light cure unit Woodpecker LED-C (Guilin Tucano Medical Apparatus and Instruments Limited Company, Guilin, China), curing light output: 850 W/cm^2^ wavelength: 420–480 nm. The samples were placed in saline (Croatian Institute of Transfusion Medicine, Zagreb, Croatia) mixed with the same amount of oral cavity moisturizer (Certmedica International GmbH, Aschaffenburg, Germany) for 14 and 28 days, respectively, at 37 °C. Every 48 h, coronal surfaces were rinsed with 200-ppm NaF solution. After the incubation period, all samples were cut with IsoMet 1000 Precision Cutter (Buehler, Lake Bluff, IL, USA) perpendicular to the joint of the material, in the mesio-distal direction. All the tests were performed two times: after 14 and 28 days of incubation, respectively.

### 2.2. Vickers Microhardness Measurement

The microhardness of samples was determined using the Qness—Q10 M—Microhardness Tester (ATM Qness GmbH, Golling an der Salzach, Austria) using the Vickers method. This method is based on observing the dentine’s resistance to plastic deformation. After the incubation period, microhardness was measured on both sides of the specimen. Two values were obtained for each specimen. The measurement was performed using 100 g (HV0.1) for 10 s. Three indents were made on each specimen, and the mean value was calculated. The spacing between the indents was at least three times their diameter. Indents were made in coronal dentine, no further than 200 μm from the material-dentine junction.

### 2.3. SEM Analysis

SEM analysis was performed on one specimen for each material, after 14 and 28 days, using a Phenom XL Scanning Electron Microscope (Phenom-World BV, Eindhoven, The Netherlands). The acquisition parameters were: 15 KV accelerating voltage, BSD Full detector, 60 Pa low vacuum, and 3840 × 2160 scan size. Before the examination, the samples were sputtered with a 10-mm-thick layer of gold. The surfaces of the sample, as well as the junction between the material and dentine, were observed.

### 2.4. EDS Analysis

EDS analysis was also conducted on one specimen for each material. The tests were made after 14 and 28 days, this time using the Inca 350 EDS System (Oxford Instruments, High Wycombe, UK). Before the examinations, the samples were gently polished with a soft brush and air-dried.

### 2.5. Statistical Analysis

The results were analyzed using SPSS 22.0 statistical software (IBM, Armonk, NY, USA). The values of materials and time points were compared using analysis of variance (repeated measures) across different materials and a Scheffe post hoc test to compare groups. The statistical significance level was set to 0.05. Before analysis, the Kolmogorov–Smirnov test was performed on the distributions, and it was established that they do not significantly differ from normal values.

## 3. Results

The results of microhardness testing in the zone that was covered with nail varnish and was unaffected by the protocols averaged at 66 ± 1.95 (HV0.1) in both testing periods and did not differ significantly among the groups (*p* > 0.05). The mean microhardness values obtained after 14 and 28 days in the zone submitted to the protocols are shown in Table 2.

The mean microhardness values (HV0.1) obtained after 14 days were significantly different between most groups (*p* < 0.001), with several exceptions (Biodentine vs. Cention Forte *p* = 0.08, Biodentine vs. Riva SC *p* = 0.997, Riva Self Cure vs. Cention Forte *p* = 0.229). Similarly, after 28 days, there were statistically significant differences between all groups (*p* < 0.001), except EQUIA Forte HT and GC Fuji TRIAGE (*p* = 0.514) and Cention Forte and Riva Self Cure (*p* = 0.687).

**Figure 1:**
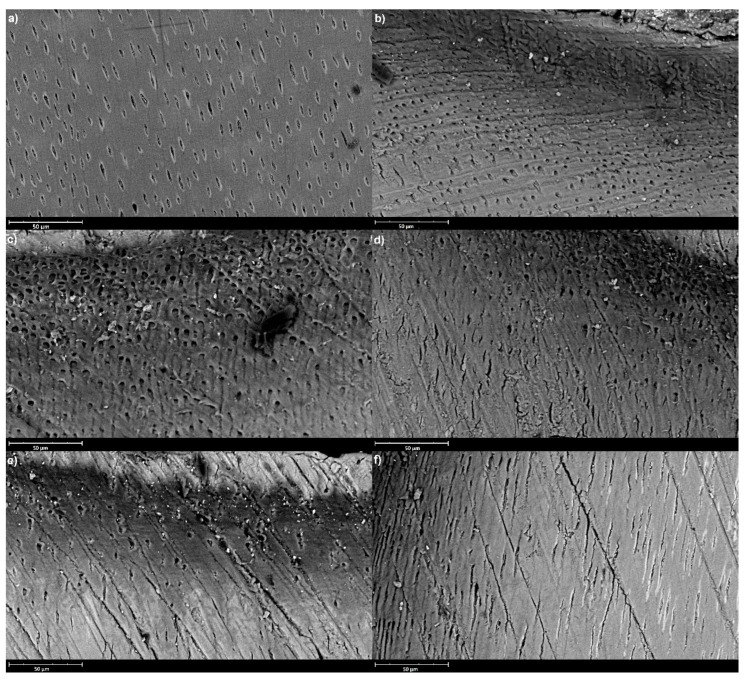
Representative SEM images (accelerating voltage of 15 kV, working distance of 8997 mm, magnification of 2100×, scale bar represents 50 μm) of the sample surfaces after 14 days of incubation; (**a**) Control, (**b**) EQUIA Forte HT, (**c**) GC Fuji TRIAGE, (**d**) Biodentine, (**e**) Riva Self Cure, (**f**) Cention Forte.

**Figure 2:**
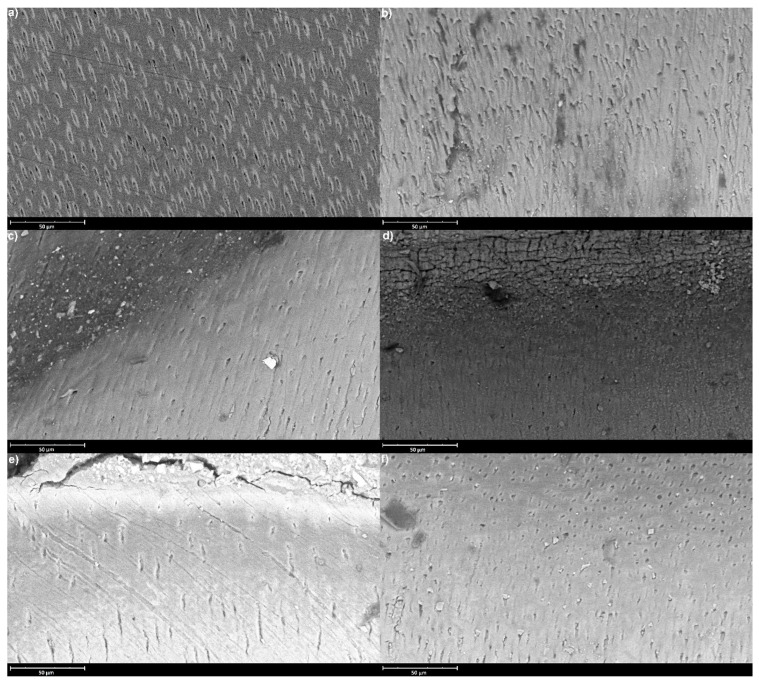
Representative SEM images (accelerating voltage of 15 kV, working distance of 8997 mm, magnification of 2100×, scale bar represents 50 μm) of the sample surfaces after 28 days of incubation; (**a**) Control, (**b**) EQUIA Forte HT, (**c**) GC Fuji TRIAGE, (**d**) Biodentine, (**e**) Riva Self Cure, (**f**) Cention Forte.

SEM analysis showed uneven patterns, mineral deposits, debris and cracks on the sample’s surfaces. Its results are shown in Figure 1 and Figure 2, which represent sample surfaces treated with different examined materials, obtained after 14 and 28 days of incubation.

**Figure 3:**
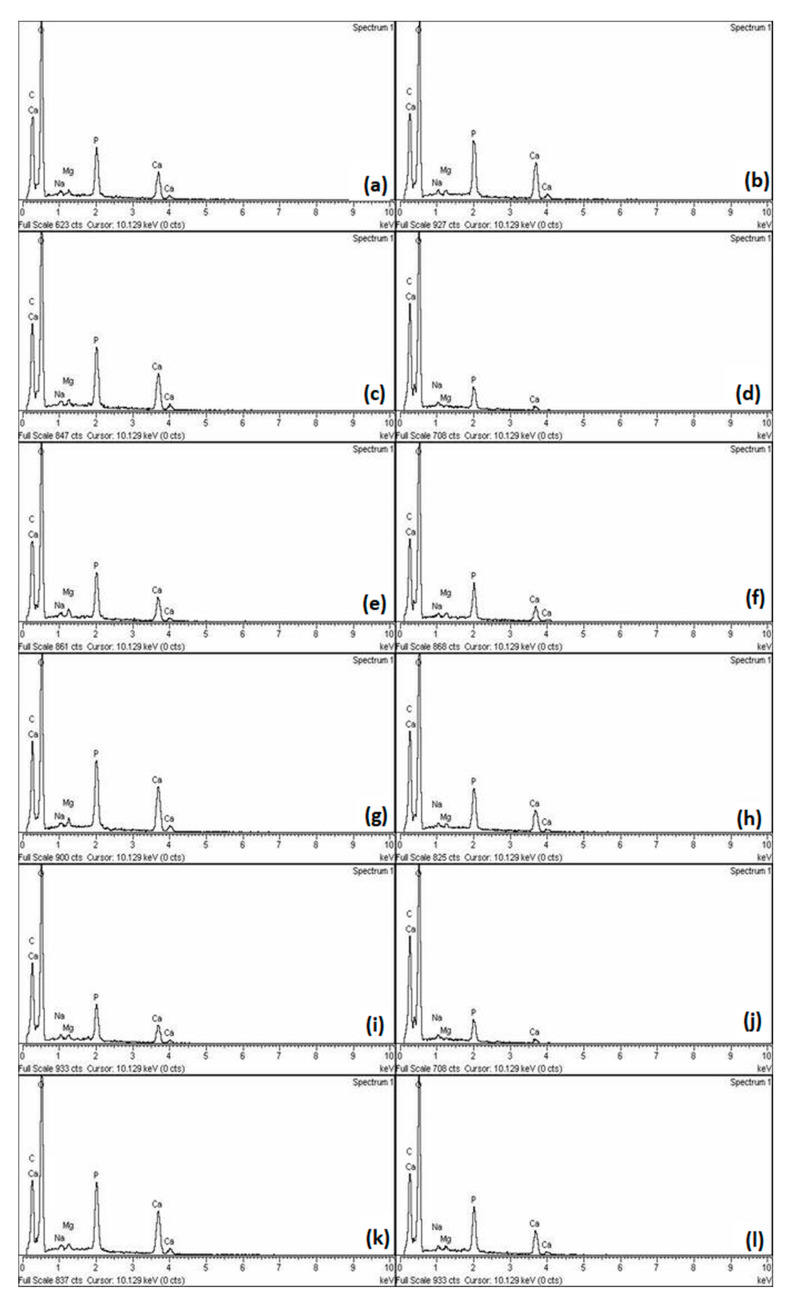
Representative results of EDS elemental analysis after 14- and 28-day incubation period of the samples in (**a**,**b**) the control group, (**c**,**d**) EQUIA Forte HT, (**e**,**f**) GC Fuji TRIAGE, (**g**,**h**) Biodentine, (**i**,**j**) Riva Self Cure and (**k**,**l**) Cention Forte.

Figure 3 shows the results of EDS analysis for each group after 14 and 28 days of incubation, with significant element share differences.

## 4. Discussion

Given the vast number of commercially available products marked as ion-releasing, in this study, we wanted to contrast more recently introduced materials with standard ones. Since the results of the present study showed significant differences among the microhardness values obtained for the tested materials, the first null hypothesis was rejected.

In the present study, the material that performed the best in microhardness testing is EQUIA Forte HT, a glass hybrid, closely followed by GC Fuji TRIAGE, a glass ionomer cement. This was further proven by the EDS analysis, which was used for observing the changes in the mineral content of the samples. EDS analysis gave an insight into the chemical changes that occurred during the demineralization and incubation processes. Following demineralization, a lower share of calcium and phosphate ions was detected, and concentrations were observed, as is expected when mineral loss occurs. On the other hand, in all five groups, increased calcium and phosphate amounts could be observed when analyzing the EDS results after incubation: the highest was in the EQUIA Forte HT group. The possible explanation for this is that due to increased matrix cross-linking, glass hybrids release ions somewhat slower, which eventually results in deeper remineralization of the lesion and higher microhardness values. However, Schwendicke et al. [24] previously did not obtain any mineral gain after the usage of GICs, most likely due to differences in the study design; in the present study, a simpler demineralization protocol was used. Furthermore, the remineralization period in our study was shorter, not allowing precipitated ions to wash off. Several factors possibly contributed to glass hybrid and glass ionomer cements performing well. Glass ionomers are already a well-known remineralizing agent, and our results are similar to others’ findings [25,26]. However, in the present study, two glass hybrids performed differently, with much higher values obtained for EQUIA Forte HT. compared to Riva Self Cure. This situation is not unprecedented: Bueno et al. [27] found that not all materials release fluorides and other ions in the same way. These differences are mostly attributed to their composition and the kinetics of their setting reaction [28], as fluoride gets easily trapped within the matrix. The diffusion may vary depending on the material composition and the extent of the reaction, which eventually determines the number of ions released [29]. In contrast, Biodentine also increased microhardness, possibly because it contains particles of a small size of 2811 m^2^/g for a quick release of calcium and phosphate ions [30], but its results were inferior to EQUIA Forte HT and GC Fuji TRIAGE, which was furthermore supported by the findings of Al-Abdi et al. [31]. However, it should be considered that this is a material that increases TGF-β1 pulp cell secretion in cell differentiation and mineralization, thus making it more suitable for in vivo studies [32]. The lowest level of mineral gain was observed in the Cention Forte group. Still, it also increased the mineral share to a certain extent. This can be explained by its properties of a low monomer conversion and lower cross-linking density of the resin matrix, which can enhance fluoride diffusion and which have possibly contributed to this result [33,34]. On the other hand, its inferior results to glass hybrids and glass ionomers are possibly due to self-cured materials releasing fluorides at a higher rate than dual-cured materials [35].

SEM/EDS analysis was performed to observe the changes in mineral content. SEM is useful for observing changes in surface structure and appearance, whereas EDS is used for observing the mineral share rise or fall [36]. Taking into consideration that the differences in the appearance of the surface structure and the mineral content of the tested samples after incubation were significant, the second and third null hypotheses were partially rejected. Samples included in this part of the study were very fragile; to reduce the examiner’s influence on the outcome, they were only gently polished with a soft brush and air-dried before SEM/EDS, leaving markings made by the saw during the cutting process visible. Furthermore, uneven accumulations of minerals can be seen on all specimens, proving the ion deposition. They penetrate to different extents into the lesion. These differences are most likely due to the unique and complex features of dentine, as its properties vary greatly through the tooth. The assessment of microhardness is widely recognized as a straightforward, dependable, and non-destructive methodology, affording valuable insights into the dynamic processes of demineralization and remineralization [37]. Previous research has proven it to be useful in relation to both dentine and enamel for observing alterations in the mechanical properties of dental tissues, allowing for the characterization of changes occurring at the microstructural level; thus, it was used in the present study [38,39]. In addition, a correlation between the mechanical properties of dental hard tissue and its mineral content was found, making this method useful for determining possible remineralization [34,40,41,42]. An artificial demineralized lesion was created using a demineralizing solution, which was suggested by Ten Cate et al. [22]. Within the limitations of this study, this was seen as sufficient, since it has been shown that an absolute simulation of oral conditions is hardly achievable, as numerous factors, including the velocity of saliva flow, its buffering capacity, dynamic pH fluctuations in the oral cavity, and behavioral variations, collectively exert substantial influence on an authentic oral environment [43,44]. As expected, the exposure of dentine to the demineralization protocol decreased the microhardness value, which is shown in the control group. As far as the duration of the remineralization course is concerned, various timespans have been used: Bertassoni’s protocol lasted several hours [19], while Talwar’s protocol lasted 28 days [45]. In the present study, microhardness was measured after 14 and 28 days to obtain results comparable to other studies. All tested materials showed an increase in microhardness in both measuring periods, with higher values obtained after 28 days. This can be explained by the gradual fluoride release, as fluoride ions do not react chemically during the setting reaction, which allows them to diffuse down their concentration gradient [46].

While all the tested materials helped to improve the dentine microhardness, compared to demineralized lesions, the obtained values were still significantly lower than those obtained on the unaffected side. This could be attributed to the fact that pulpal defense and the activity of its cells, such as odontoblasts and fibroblasts is, with the presence of growth factors, crucial for functional mineral deposition [47], which is another limitation of the study. Still, the examined materials, with their ability to deploy ions, can be a valuable clinical asset, due to their buffering effect and anti-microbial effects [48,49]. As the remineralization of dentine is very complex and often results in the heterogenous formation of crystals, great differences between studies are possible, making comparisons challenging. However, such in vitro studies, in which the oral conditions were not utterly simulated, still gave us an idea of the materials’ capability for releasing ions, which, together with the pulpal activity, can lead to remineralization. Nevertheless, the results are to be observed while considering the experimental conditions. Therefore, additional research is needed to answer a great number of questions that arise regarding this fast-developing group of materials.

## 5. Conclusions

Within the limitations of this study, EQUIA Forte HT, a glass hybrid, showed the highest potential for the release of ions into an artificially demineralized lesion, as evidenced by its effect on the microhardness and chemical composition when compared to Riva Self Cure, Cention Forte, Biodentine and GC Fuji TRIAGE. However, all materials tested increased the mineral content of the lesion, making the use of ion-releasing materials promising, along with further research that could include longer exposure periods and different environmental conditions. Finally, conducting comprehensive studies to evaluate the biological responses and clinical outcomes associated with these materials would provide valuable insights to support the effective use of these materials.

## Figures and Tables

**Table 1:** Materials used in the study.

Material	Type	Manufacturer
EQUIA Forte^®^ HT	Glass hybrid	GC Corporation, Tokyo, Japan
Riva Self Cure	Glass hybrid/glass ionomer cement [21]	SDI Limited, Melbourne, VI, Australia
Cention Forte	Alkasite	Ivoclar Vivadent AG, Schaan, Liechenstein
Biodentine™	Tricalcium silicate-based material	SEPTODONT, Saint-Maur-des-fossés Cedex, France
GC Fuji TRIAGE^®^	Glass ionomer cement	GC Corporation, Tokyo, Japan

**Table 2:** Mean dentine microhardness and SD values (HV0.1) after 14 and 28 days (*p* < 0.001).

Material	After 14 Days	After 28 Days
EQUIA Forte^®^ HT	26.7 ± 1.45	37.74 ± 1.56
Riva Self Cure	19.66 ± 1.02	29.58 ± 1.18
Cention Forte	19.01 ± 1.24	27.93 ± 1.33
Biodentine™	23.35 ± 1.23	29.92 ± 1.02
GC Fuji TRIAGE^®^	25.94 ± 1.35	33.87 ± 5.57
Control Group	15.57 ± 0.68	15.64 ± 0.82

## Data Availability

The data presented in this study are available on request from the corresponding author.
